# Selective Decontamination of the Digestive Tract in Hepatobiliary Surgery: A Concept

**DOI:** 10.1155/1990/65141

**Published:** 1990

**Authors:** M. J. H. Slooff, C. Rosman, D. Van Der Waaij

**Affiliations:** 1 Departments of Surgery and Microbiology Academisch Ziekenhuis Groningen The Netherlands; 2 Academisch Ziekenhuis Department of Surgery Oostersingel 59 Groningen 9713 EZ The Netherlands


HPB Surgery, 1990, Vol. 2, pp. 1-5
Reprints available directly from the publisher
Photocopying permitted by license only

© 1990 Harwood Academic Publishers GmbH
Printed in the United Kingdom

### REVIEW

### SELECTIVE DECONTAMINATION OF THE DIGESTIVE TRACT IN HEPATOBILIARY SURGERY: A CONCEPT

M.J.H. SLOOFF*, C. ROSMAN and D. VAN DER WAAIJ

*Departments of Surgery and Microbiology, Academisch Ziekenhuis Groningen, the Netherlands*

*(Received 6 August 1989)*

Infection is a common problem in hepatobiliary surgery. Keighly and Lewis et al. reported infected bile in 31–33% of patients after an operation for biliary disease. Such patients had an increased risk of post-operative infections compared to patients with sterile bile[1,2]. These findings are confirmed by reports from Flemma et al. and Maddocks et al.. They found that bacteria are common in bile under pathological conditions like stones in the common bile duct, the presence of strictures especially partial strictures or cholangitis[3,4]. Keighly and Blenkhard also reported a strong relation between per-operative bile cultures and cultures from wound abscesses and blood after surgery on the biliary tract in patients with infected bile. In 64% of the wound abscesses and in 90% of the episodes of bacteraemia the same micro-organisms were observed as had previously been identified in the bile[5]. Micro-organisms frequently found in these cultures are gram-negative bacteria like E coli, Klebsiella spp or Pseudomonas spp. Sometimes gram-positives are found like Streptococcus faecalis. In patients with a biliodigestive anastomosis anaerobes may be found[2,5]. Intra-abdominal infections after liver resection are common. Incidences varying between 8 and 30% have been reported from different institutions[6,7,8]. Moreover, in liver transplant programs infection is still the major cause of morbidity and mortality[9,10,11]. Therefore, the possible mechanisms of infection of the hepatobiliay system should be one of the major interests in hepatobiliary surgery.

Bacteria may enter the liver and biliary tract in two ways. Either directly via the transsphincteric route or indirectly by translocation through the intestinal mucosa via the portal blood or the lymphatic system[12,13], In both instances the gastro-intestinal tract is the source of the invading micro-organisms.

Normally, the upper part of the gastro-intestinal tract contains only few bacteria. This is due to the combined influence of acid secretion of the stomach and the inhibitory action of bile and pancreatic juices on many micro-organisms. The majority of the bacteria present are gram-negative cocci and baccilli. Occasionally,

*Address for correspondence: Dr. M.J.H. Sloof, Academisch Ziekenhuis, Department of Surgery, Oostersingel 59, 9713 EZ Groningen, The Netherlands.

enterococci lactobacilli and diphteroid bacteria are found[14]. Bile samples from patients with normal bile ducts are sterile[15]. Any obstruction of the bile flow in the biliary tract can interfere with the normal clearing mechanism and thus enhance bacterial colonisation of this system[3]. Moreover, some miocro-organisms are apparently able to tolerate high concentrations of bile acids and colonise the biliary system in case of obstructions. Such bacteria are Salmonella, Enterococcus, Clostridium and Bacteroides fragiles[5,16]. Translocation is the other major factor leading to infection of the liver and biliary tract. Endogenous intestinal bacteria may translocate to the liver and biliary tract under certain abnormal conditions. These conditions are generated during long anaesthesia, extensive abdominal trauma or cancer radiotherapy or chemotherapy[17,18,19,20]. All these factors interfere with intestinal motility, which is one of the important factors controlling bacterial overgrowth in the gut[21]. When potential pathogenic bacteria grow out in large numbers they translocate from the intestinal lumen to the liver and spleen [12,16]. In addition overgrowth of gram-negative bacteria may finally lead to an increase in the intestinal concentration of endotoxins, which are apparently promotors of translocation[22].

In a normal liver the Kupffer cells, as part of the reticulo-endothelial system, clear the portal blood of intestinal bacteria and endotoxins[23].However, in pathological conditions such as juandice or a newly transplanted liver graft, the function of the Kupffer cells may be compromised resulting in a diminished clearing capacity for the portal blood[13,24,25]). Consequently, it is possible that the biliary tract might be colonised from within the liver under certain pathological conditions. The early experimental work of Dineen has provided proof of such a mechanism[26]. He was able to show in a pig model that colon bacilli injected into the portal system via the spleen could be subsequently cultured in the bile. This concept concerning the origin of hepatobiliary infection from inside the intestinal lumen either directly, trans-sphincterically or indirectly via translocation has also been postulated by others [11,24,27,28].

The normal colonisation pattern of the small and large intestines is controlled and maintained by host factors as well as by their indigenous micro-flora[29]. Important host factors are: intact anatomy, intestinal motility, mucus secretion, desquamation of epithelial cells from the villi and secretion of immunoglobulin A. These factors act in addition to the low pH of the stomach and the antimicrobial activity of the bile itself. The strongest influence, however, is exerted by the indigenous flora. Not only does the indigenous flora compete successfully with newly ingested bacteria, it is also involved in the production of substances which stimulate peristalsis, smooth muscular tension and mucus secretion [30]. This intestinal mucus functions not only as a conveyor belt for the intestinal contents, but also as a feeding layer for the indigenous flora. This network of interrelated activities between host and indigenous flora provides a very strong protection against colonisation of the intestinal tract by pathogenic bacteria. This protective mechanism, called colonisation resistance (CR) of the digestive tract, has recently been comprehensively reviewed [31].

It is obvious that the CR is impaired if either host factors contributing to the CR, such as peristalsis, are disturbed or when the indigenous flora is significantly modified, i.e. by antibiotic treatment. This results invariably in "overgrowth" with potentially pathogenic bacteria.

From the preceeding it is clear that in patients with biliary obstruction or impaired liver function "overgrowth" of intestinal bacteria may already be present before surgery. The operation by itself may enhance this phenomenom not only by the induced intestinal paralysis, but also as a result of manupilation of the intestines, liver or bile ducts. Any action aimed at a reduction of the infection incidence in such patients should be focussed on the correction of "overgrowth" of pathogenic bacteria and its effects such as translocation. To achieve this aim measures can be taken to alter the actual flora and attempts can be made to restore the CR before the operation. A possible measure could be selective decontamination of the digestive tract (SDD) as proposed by van der Waaij (29) and recently reviewed by Clasener et al [32]. SDD is induced by means of oral small spectrum anti gram-negative antibiotics and occasionally combined with parenteral antibiotics. These antibiotics have been selected for NOT being effective against the greater part of gram-positive predominantly anaerobic indigenous intestinal flora. The anaerobic flora is kept intact, because of its value for the CR of the gastro-intestinal tract. Few antibiotics have been found suitable for SDD [29]. In our unit SDD is started two days before elective operations with orally administered doses of the non-absorbable antibiotics: polymyxin E (4 times 100 mg daily), tobramycin (4 times 80 mg daily) and amphotericin B (4 times 500 mg daily). In non-elective cases the regimen is started directly before surgery. Postoperatively selective decontamination of the oral cavity is maintained by a sticky paste (orobase) containing polymyxin E 2%, tobramycin 2% asnd amphotericin B 2%. This paste is applied to the oral mucosa four times daily. The SDD of the gut is continued with the use of the same antibiotics as given pre-operatively. The drugs are administered via the nasogastric tube which is subsequently closed for one hour. Because it takes some time for this regimen to take effect in killing gram-negative potentially pathogenic bacteria and yeasts, parenteral antibiotics are started at the induction of anaesthesia and continued for 48 hours. Mostly tobramycin 3 times daily 80 mg and a cephalosporine are used.

The validity of this SDD-concept as a measure to prevent gram-negative infections has not yet been proven in proper randomized clinical trials in hepatobiliary surgery. However, SDD has been proven to be effective in several categories of patients other than patients with hepatobiliary disease. Sleijfer and co-workers reported on their experience with such a regimen in patients who were severely granulocytopenic after chemotherapy for cancer treatment [33]. In his study a significantly lower gram-negative infection rate and a significantly lower mortality due to infection was observed in patients treated with SDD compared to patients treated without SDD. These observations are in accordance with finding of Gurwith et al. and Rozenburg et al. [34,35]. Stoutenbeek and co-workers reported on the incidence of infections in the intensive care unit in trauma patients with and without SDD. Fifty-nine patients constituted a historical control group and had no SDD. The gram-negative infection rate in this group was 81% compared to 16% in patients (N = 63) who had SDD [36]. In a prospective study Kerver was also able to prove the beneficial effects of SDD on the reduction of gram-negative infections and mortality rate in an intensive care unit [37]. Such observations are confirmed by others. Ledingham *et al.* showed a consistent reduction in colonisation of the digestive tract with aerobic gram-negative baccilli with a concomitant reduction of the incidence iof acquired infections when SDD was used. In addition, the mortality rate in certain categories of patients was reduced too [39].

The liver transplant group of our hospital uses SDD for infection prevention. If

SDD is instituted effectively, meaning that fewer than 10 gram-negative microorganisms are found in one gram of faeces, significantly fewer gram-negative infections are observed compared to patients with an unsuccessful SDD regimen [39]. Also Wiesner and co-workers from the Mayo Clinics were able to show the effectiveness of an identical SDD regimen for infection prevention in liver transplant patients [11].

The central problem in patients with hepatobiliary disease is twofold. The CR is already disturbed before the operation due to the absence of diminished concentration of bile in the gut. Additionally, there exists a decreased capacity of Kupffer cells to clear the portal blood from tanslocated bacteria and endotoxins. The SDD regimen instituted some time before an operation may restore the normal colonisation pattern of the gut as far as potentially pathogenic bacteria are concerned thereby reducing overgrowth and thus diminishing translocation via the portal blood and lymphatics. To remain effective after the operation the SDD-treatment should be continued, because factors like bowel paralysis, external biliary drainage and liver dysfunction endanger the normal CR and may stimulate further translocation. The SDD can be discontinued when normal bile flow is restored and normal bowel motility is present and wounds are healed.

*Acknowledgements*

We gratefully acknowledge the critical comments of Drs. I.J. Klompmaker and Dr. A.J.H. Kerver and the editorial assistance of Mrs M.L.B. Schaub-Gorsira

*References*

1. Keighley MRB. (1977) Micro-organisms in the bile. A preventable cause of sepsis after biliary surgery. *Ann. Roy. Coll. Surg. Eng.* **59**, 328–334
2. Lewis RT, Goodall RG, Marien B, Park M, Lloyd-Smith W, Wiegand FM. (1987) Biliary bacteria, antibiotic use, and wound infection in surgery of the gallbladder and common bile duct. *Arch.Surg.* **122**, 44–47
3. Flemma RJ, Flint LM, Oosterhout S, Shingleton WW. (1967) Bacteriologic studies of biliary tract infection. *Ann.Surg. 166*, **6**, 563–572
4. Maddocks AC, Hilson GRF, Taylor R. (1973) The bacteriology of the obstructed biliary tree. *Ann Roy Coll Surg Engl*, **52**, 316–319
5. Keighley MRB, Blenkhard JI. (1988) Infection and the biliary tree. In: Surgery of the liver and biliary tract. Ed.Blumgart LH. Edinburgh, Churchill Livingstone: pp.121–132
6. Iwatsuki S, Shaw BW, Starzl TE. (1983) Experience with 150 liver resections. *Ann Surg*, **197**, 247–253
7. Thompson HH, Tompkins RK, Longmire WP. (1983) Major hepatic resection. A 25-year experience. *Ann Surg* **197**, 375–388
8. Ong GB, Lee NW. (1975) Hepatic resection. *Br J Surg* **62**, 421–430
9. Cuervas-Mons V, Martinez AJ, Dekker A, Starzl TE, van Thiel DH. (1986) Adult liver transplantation: An analysis of the early causes of death in 40 consecutive cases. *Hepatology 6*, **3**, 495–501
10. Rubin R. (1988) Infectious disease problems. In: Transplantation of the liver. Ed.Maddrey WC. New York, Elsevier: pp 279–309
11. Wiesner RH, Hermans PE, Rakela J, Washington II JA, Perkins JD, DiCecco S, Krom RAF. (1988) Selective bowel decontamination to decrease gram-negative aerobic bacterial and candida colonization and prevent infection after orthotopic liver transplantation. *Transplantation* **45**, 570–574
12. Berg RD, Carlington AW. (1979) Translocation of certain indigenous bacgeria from the gastrointestinal tract to mesenteric lymphe nodes and other organs in a gnotobiotic mouse model. *Infect Immun* **23**, 403–411

13. Rush BF, Sori AJ, Murphy TF, Smith S, Flanagan JJ, Machiedo GW. (1988) Endotoxemia and bacteremia during hemorrhagic shock. The link between trauma and sepsis? *Ann Surg 207*, 5, 549–552
14. Bernhardt H, Knoke M. (1980) Characterization of the microflora of the small intestine. *Zbl Bakt Hyg I Abt A* **246**, 379–392
15. Nolan JP. (1978) Bacteria and the liver. *New Engl J Med* **299**, 1069–1070
16. Van der Waaij D, Berghuis- de Vries JM, Lekkerkerk- van der Wees JEC. (1972) Colonisation resistance of the digestive tract and spread of bacteria to the lymphatic organs in mice. *J Hyg* **70**, 335–342
17. Deitch EA, Bridges W, Baker J et al. (1988) Hemmorrhagic shock-induced bacterial translocation is reduced by xanthine oxidase inhibition or inactivation. *Surgery 104*, 191–198
18. Berg RD. (1983) Bacterial translocation from the gastrointestinal tract of mice receiving immunosuppressive chemotherapeutic agents. *Current Microbiol* **8**, 285–292
19. Wells CL, Maddans A, Simmons RL. (1988) Proposed mechanism for the translocation of intestinal bacteria. *Rev Inf Dis* **10**, 958--979
20. Deitch EA, Winterton, J. Li M et al. (1987) The gut as portal of entry for bacteremia. Role of protein malnutrition. Ann Surg 205, 681–692
21. Sykes PA, Boulter KH, Schofield PF. (1976) Alterations in small-bowel microflora in acute intestinal obstruction. *J Med Microbiol* **9**, 13–22
22. Deitch EA, Berg RD, Specian R. (1985) Endotoxin but not malnutrition promoted bacterial translocation of the gut flora in burned mice. *Trauma* **27**, 161–166
23. Drivas G, James O and Wardke N. (1976) Study of reticuloendothelial phagocytic capacity in patients with cholestasis. *Br Med J* 31, 1568–1569
24. Fine J. (1965) Current status of the problem of traumatic shock. *Surg Gynecol Obstet* **120**, 537–544
25. Roughneen PT, Kumar SC, Pellis NR, Rowlands BJ. (1988) Endotoxemia and cholestasis. Surg Gynecol Obstet 167, 205–210
26. Dineen P. (1964) The importance of the route of injection in experimental biliary tract obstruction. *Surg Gynecol Obstet* **119**, 1001
27. Marshall JC, Christou NV, Horn R, Meakins JL. (1988) The microbiology of multiple organ failure. The proximal gastrointestinal tract as an occult reservoir of pathogens. *Arch Surg* **123**, 309–315
28. Wajszczuk CP, Dummer JS, Ho M, van Thiel DH, Starzl TE, Iwatsuki S, Shaw Jr B. (1985) Fungal infections in liver transplant recipients. *Transplantation* **40**, 4, 347–353
29. Van der Waaij D. (1982) Colonization resistance of the digestive tract: clinical consequences and implications. *J Antimicrob Chemother* **10**, 263–270
30. Gordon HA, Brucker-Kardos E. (1961) Effect of normal flora on intestinal surface area. *Am J Physiol* **201**, 175–178
31. Van der Waaij D. (1989) The ecology of the human intestine and its consequences for overgrowth by pathogens such as *Clostridium difficile*. *Ann Rev Microbiol* **43**, 69–87
32. Clasener HAL, Vollaard EJ, Van Saene HKF. (1987) Long-term prophylaxis of infection by selective decontamination in leukopenia and mechanical ventilation. *Rev Inf Dis* **9**, 295–328
33. Sleijer DTh, Mulder NH, de Vries-Hospers HG, Nieweg HO et al. (1980) Infection prevention in granulocytopenic patients by selective decontamination of the digestive tract. *Eur J Cancer* **16**, 859–869
34. Gurwith MJ, Brunton JL, Lank BA, Harding GKM, Ronald AR. (1979) A prospective controlled investigation of prophylactic trimethoprim/sulfamethoxazole in hospitalized granulocytopenic patients. *Am J Med* **66**, 248–256
35. Rozenberg-Arska M, Dekker AW, Verhoef J. (1983) Colistin and trimethoprim-sulfamethoxazole for prevention of infection in patients with acute nonlymphocytic leucemia. Decrease of emergence of resistant bacteria. *Infection* **11**, 167–169
36. Stoutenbeek ChP. (1987) Infection prevention in intensive care. Infection prevention in multiple trauma patients by selective decontamination of the digestive tract (SDD). Thesis. ISBN 9001736–4
37. Kerver AJH. (1988) Nosocomial infections and infection prevention in surgical intensive care patients. Thesis. ISBN 90-9002254-6
38. Ledingham IM, Eastaway AT, McKay IC, Alcock SR, McDonald JC, Ramsay G. (1988) Triple regimen of selective decontamination of the digestive tract, systemic cefotaxime, and microbiological surveillance for prevention of acquired infection in intensive care. *Lancet*, **April 9**, 785–790
39. Rosman C. Unpublished data

